# Thermally-Induced Lactosylation of Whey Proteins: Identification and Synthesis of Lactosylated β-lactoglobulin Epitope

**DOI:** 10.3390/molecules25061294

**Published:** 2020-03-12

**Authors:** Alessandra Gasparini, Sofie Buhler, Andrea Faccini, Stefano Sforza, Tullia Tedeschi

**Affiliations:** 1 Food and Drug Department, University of Parma, Parco Area delle Scienze 27/A, 43124 Parma, Italy; alessandra.gasparini@studenti.unipr.it (A.G.); sofie.buhler@yahoo.it (S.B.); stefano.sforza@unipr.it (S.S.); 2 Centro Interdipartimentale Misure (CIM), University of Parma, Parco Area delle Scienze 27/A, 43124 Parma, Italy; andrea.faccini@unipr.it

**Keywords:** lactosylation, whey proteins, peptide synthesis, IgE-binding epitopes

## Abstract

The high temperatures used in the production of milk may induce modifications in proteins structure. Due to occurrence of the Maillard reaction, lactose binds lysine residues in proteins, affecting the nutritional value. Milk is also an important source of allergenic proteins (i.e., caseins, β-lactoglobulin and α-lactalbumin). Thus, this modification may also affect the allergenicity of these proteins. Focusing on milk whey proteins, a screening on different Ultra High Temperatures (UHT) and pasteurized milk samples was performed to identify lactosylation sites, in particular in protein known epitopes, and to verify the correlation between lactosylation and the harshness of the treatment. Whey proteins were extracted from milk samples after caseins precipitations at pH 4.6 and, after chymotryptic and tryptic in solution digestion, peptides were analysed by UPLC-MS and LTQ-Orbitrap. Results show the presence of lactosylated lysine residues in several known epitopes. Then, a β-lactoglobulin epitope was selected and synthesized by solid phase synthesis followed by in solution lactosylation, obtaining high reaction yields and purities. The synthesis of lactosylated allergenic epitopes, described here for the first time, is a useful tool for further studies on the technological impacts on food allergenicity.

## 1. Introduction

**Figure 1:**
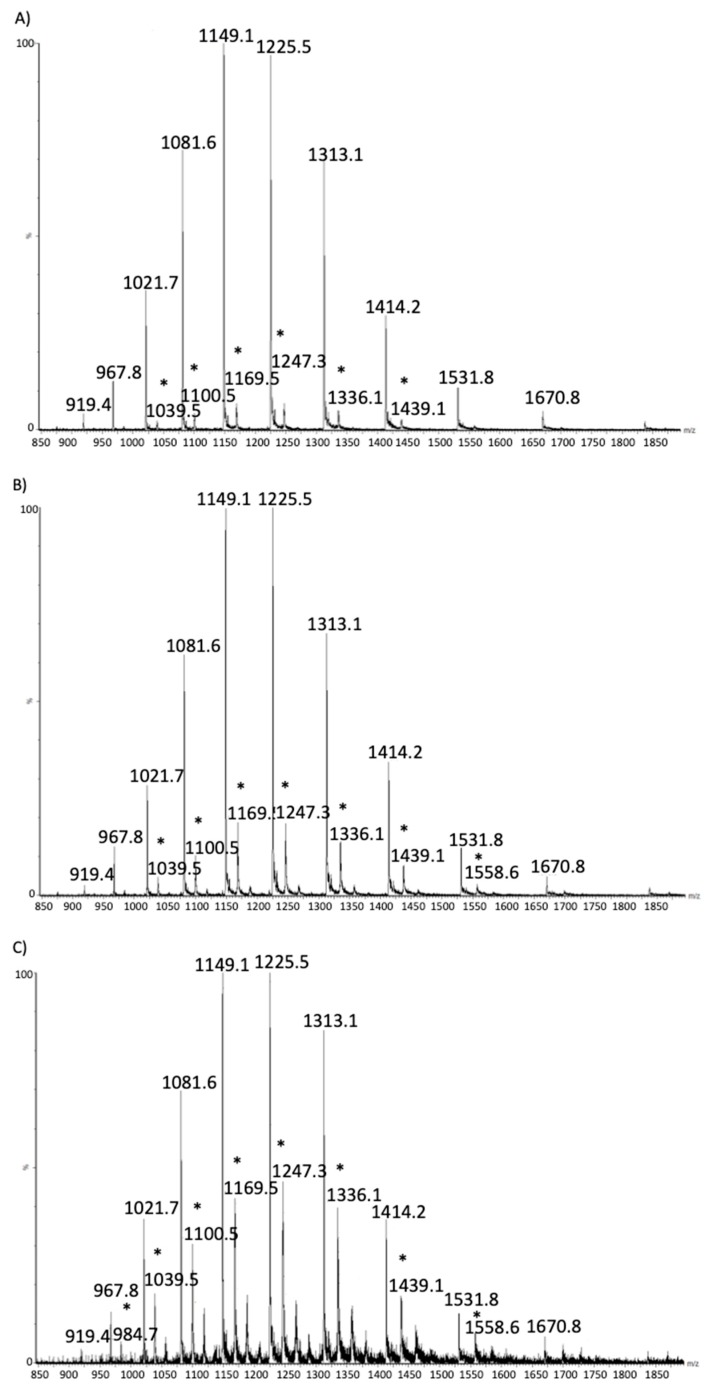
β-lactoglobulin. MS spectrum from raw (**A**), pasteurized (**B**) and Ultra High Temperature (UHT) samples (**C**). Star highlights MS ions corresponding to the lactosylated form of the proteins. Intensity is on the y axis, while *m*/*z* on the x axis.

Before commercialization, milk undergoes different technological treatments in order to guarantee its microbiological safety and extended shelf life [1]. Main thermal treatments used are pasteurization (15–20 s at 72–75 °C), sterilization (10–30 min more than 110 °C) and Ultra High Temperature (UHT) treatment [1]. The UHT treatment may be performed by direct heating, through steam injection (2–4 s at 140–145 °C) or by indirect heating, via metal plates or tubes (5–8 s at 136–138 °C) [2,3]. The high temperatures involved in the treatment affect the proteins, inducing some relevant structural and chemical modifications, such as protein denaturation/aggregation and the Maillard reaction [4]. This reaction mainly affects lysine residues that react with lactose forming lactulosyllysine (Amadori compound), as shown in Figure 1.

The main effect of the lactosylation is to reduce the available free lysine residues, affecting the nutritional value of the product itself [5]. Thus, several studies were performed in order to investigate lysine glycation of whey proteins such as β-lactoglobulin and α-lactalbumin. The most common analysis used for the identification of glycated modifications is the determination of furosine, a degradation product of the Amadori compound, by HPLC. The detection of lactosylated lysine residues in peptides and proteins is performed by LC-MS techniques and MALDI-TOF-MS considering a mass shift of +324 (the molecular weight of lactose) with respect to the unmodified protein [2,6,7,8,9]. The first lactosylation site (i.e., the lysine 100 of β-lactoglobulin) was identified in 1998 with electrospray mass spectrometry [10]. The identification of process-induced modifications may be performed with an enzymatic digestive step before the MS analytical investigation. The digestion may be performed in gel after electrophoresis or in solution, using enzymes like endoproteinase GluC, endoproteinase AspN and trypsin [6].

Moreover, N^ε^-carboxymethyllysine (CML) is also used as an indicator of the advanced stage of the Maillard reaction. For its detection different analytical technique may be used: derivatization with o-phthalaldehyde (OPA) reverse phase HPLC, GC-MS with acetylation of the amine groups and methylation of the carboxylic groups, UPLC and MALDI-TOF-MS [11,12].

The binding with lactose on lysine residues may affect not only whey proteins nutritional value, but also their allergenicity. Indeed, milk is an important source of allergenic proteins (i.e., caseins and whey proteins) and Cow’s Milk Allergy (CMA) is one of the most common food allergies affecting approximately 2–5% of newborns [13]. Among abundant whey proteins, both β-lactoglobulin and α-lactalbumin are important milk allergens [14]. The presence of lactose bound to the IgE-binding epitope could influence the interaction altering the allergenic response. With regards to β-lactoglobulin, it has been demonstrated that low or mild glycation produce no effects on the binding with IgE. On the contrary, in the presence of high rate of glycation, the binding with antibodies decreases. This finding could be related to the masking effect of the sugar on the epitope [15]. Other studies on the effects of the conjugation of sugars on the epitopes showed a decrease in antigenicity [16,17,18]. However, the molecular interaction between the lactosylated epitope and the antibody is still unknown.

Here, we describe the results of a work aimed at identify the preferential glycated sites in whey proteins in a series of milk samples, and the development of a synthetic approach for lactosylated peptides sequences involved in allergenic epitopes. As a first step, the presence of the lactosylation sites in different UHT and pasteurized milk samples were investigated to find a correlation with the harshness of the thermal treatment applied and to identify the preferential lactosylation sites. Then, the identified modification’s sites were compared with the known epitopes to verify the presence of lysine residues that could be lactosylated and thus could affect the allergic response. Finally, a protocol for the synthesis of β-lactoglobulin epitopes probes in the lactosylated form (chosen among the ones expected to be prevalent) was developed for future allergenicity studies.

## 2. Results and Discussion

### 2.1. Milk Samples Screening

A series of milk samples (seven pasteurized milk and seventeen of UHT milk) were obtained from local retailers. Samples belonged to different brands (13), and were produced in different countries (Italy, Slovenia, Germany). One sample of raw milk, without any treatment, was obtained from a local farm. The first step was to identify possible whey proteins in lactosylated form, looking for differences between the three types of product (raw, pasteurized and UHT milk). With this aim, soluble whey proteins were extracted after manual defatting and casein precipitation at pH 4.6. Solutions were then filtered and analysed by SDS-PAGE analysis. No differences were observed between the extracted wheys in the SDS-PAGE lanes, which showed the major presence of α-lactalbumin and β-lactoglobulin, while caseins were confirmed to be absent in all of them (data are shown in supporting material).

### 2.2. Whey Proteins Identification and Quantification

To identify and quantify whey proteins in the native and lactosylated forms, UPLC-MS analysis was then performed applying an analytical method recently developed by our group [19].

Briefly, despite glycated and unmodified forms of α-lactalbumin and β-lactoglobulin co-elute in the chromatogram, it is possible to identify the presence of the lactosylated forms of each protein in MS spectra. Figure 1 shows the results obtained for β-lactoglobulin isoform A from a pasteurized, a UHT and raw samples, respectively. Lactosylated forms of the whey proteins in the MS spectra are identified as an increase in protein mass of 324 Da (the lactose molecular mass) leading to a different multi-charged pattern as compared to unmodified proteins.

The amount of soluble whey proteins in the samples was quantified with the external calibration curves. Quantification was performed by integration of the eXtracted Ion Chromatograms (XIC) peaks areas as previously described [19] (Table 1).

As expected, the percentage of lactosylated protein increases with the harshness of the treatment, as already reported in the literature [7,20,21].

The fact that in all samples the monolactosylated forms are more abundant than the di- and highly lactosylated ones, indicates that the subsequent identification of the lactosylation sites will focus on the most reactive sequences which are most prone to be lactosylated.

### 2.3. Identification of Lactosylation Sites in Whey Proteins

To identify lysine residues involved in lactose binding, an in solution tryptic and chymotryptic digestion was performed on the most glycated samples, i.e., UHT milk. Beside trypsin, commonly used for these experiments due to its specificity for arginine and lysine residues, it has been decided to also use chymotrypsin, having a different specificity (aromatic amino acids residues). This additional digestion was planned considering that lysine glycosylation might affect digestibility with trypsin. Indeed, it is expected that the activity of lysine/arginine specific proteases is negatively affected by the presence of the sugar changing the charge and the size of the side chain [22]. Since the amount of lactosylated forms was too low for an accurate identification, the whey was concentrated by ultrafiltration using a cut-off of 3 kDa and keeping the retentate.

Using this approach, several lactosylated peptides were identified in the UHT milk samples. Lysine residues glycated in α-lactalbumin are reported in Table 2, whereas those in β-lactoglobulin in Table 3. A full list of identified peptides is given in Supplementary Materials.

Additional peptides carrying a sugar were detected in the digestion performed with chymotrypsin, suggesting, as hypothesized, that trypsin activity may be affected by the presence of the sugar on lysine residues. From the results, it was possible to identify the lysine residues involved in the Maillard reaction.

In regards to β-lactoglobulin using UHPLC-MS/MS/MRM mass spectrometry it has been already demonstrated, on a series of commercial milk products, that after thermal processes were identified a series of lactosylation sites (i.e., K8, K14, K47, K60, K69, K75, K77, K83, K91, K100, K101 and K135) [23]. Moreover, studies on glycation degree carried out using nRPC-ESI-MS/MS revealed the following glycation sites: K47, K60, K69, K70, K75, K77, K83, K91, K100, K101, K135, K138, K141. With regards to α-lactalbumin, K5, K13, K62, K93, K94, K98, K108, K114, K122 sites were identified [7,20]. Residues K16, K58 and K79 were also identified as possible α-lactalbumin lactosylation sites [6].

Results on glycation sites here reported (Table 2 and Table 3) further confirm literature data, except for residue K114 of α-lactalbumin and K8 and K60 of β-lactoglobulin that were not found glycated, most likely due to the low amount of these glycated peptides in our sample preparations.

Thus, the glycation sites here reported are to be considered as the prevalent glycations sites, and the first to be subjected to lactosylation when the Maillard reaction is limited. 

### 2.4. Identification of Lactosylation Sites in IgE Binding Epitopes

β-lactoglobulin is the most abundant whey protein and it is one of the first antigens appearing in human diet after lactation, being present in bovine milk but not in human one [24]. Thus, we focused our attention on this protein to identify lactosylation sites on potential epitopes. To reach this goal, chymotryptic and tryptic digested samples were analysed by UPLC-MS and results compared with those obtained by LTQ-Orbitrap analysis. Table 4 shows modified peptides identified by both techniques.

Carboxymethylation derives from the oxidative cleavage of lactulosyllysine (the Amadori compound) [25]. Data were then compared with known epitopes already described in literature [26]. 

Indeed some epitopes, listed in Table 5, have in the sequences lysine residues that are also glycation sites.

The presence of lactose bound to IgE-binding epitopes could affect the binding between the antibody and the antigen altering the allergenic response. In order to have suitable compounds to investigate the subject, among the epitopes reported in Table 5 (and also expected to be preferential lactosylation sites) the first one was selected and synthesized in the unmodified and in the lactosylated form, in order to obtain material for future studies about the effects on the binding with the IgE with ELISA tests.

### 2.5. Peptide Synthesis and Lactose Conjugation

The epitope (TKIPAVFKIDALNEN) was synthesised using the Fmoc protocol for the Solid Phase Peptides Synthesis as already described (Section 3.6). For the synthesis, a Rink Amide resin was used to obtain an amide group at the C-terminal of the sequence. Before cleavage, an additional step was performed to acetylate the N-terminal of the sequence. These strategies were used to obtain peptides able to mimic the sequences into the entire protein structure.

Since the cleavage from the resin needs acidic conditions that may remove lactose from the peptide, the lactosylation step was performed after the release from the resin. Several procedures for the glycation of whey proteins are reported in literature to investigate the effects of the presence of the sugar on the main protein properties [27,28,29]. Some studies are reported for the glycation of peptides [30,31], in particular with lactose. Starting from the literature [22], several attempts were made to define the optimal reaction conditions. Reactions were performed with the absence of any solvents, mixing together the peptide and lactose and evaporating the buffer solution by nitrogen flux. Different temperatures were tested (50, 60 and 70 °C) as well as different peptide:lactose molar ratios applied (1:4, 1:8, 1:12, 1:16, 1:24). Higher yields were obtained at 70 °C and with 1:16 ratio. A reaction time of 48 h looks better than 24 h, since a higher amount of lactosylated peptide was obtained. Reaction yields were determined by integration of the chromatographic peaks areas. Percentages of modified peptide were determined from the ratio between the XIC areas of the peptide in the modified and unmodified forms (Table 6).

**Figure 2:**
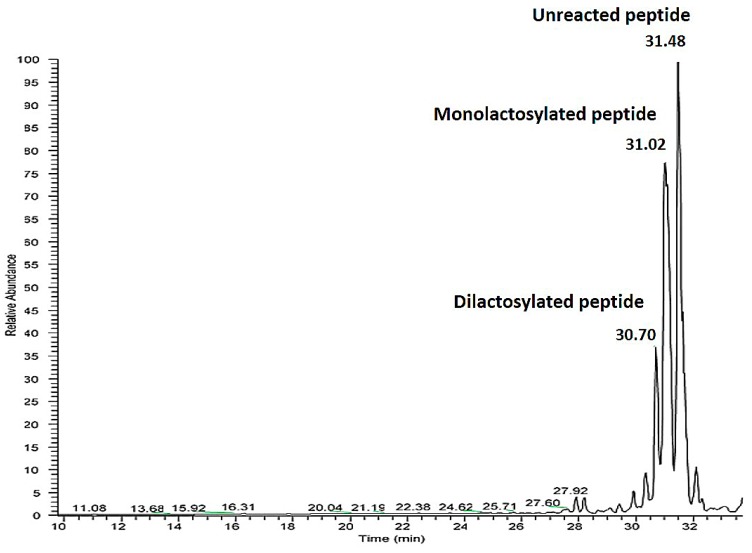
UHPLC-MS/MS analysis of the reaction mixture obtained adopting the conditions described in Table 5 line 5. TIC (total ion current) profile of reaction mixture obtained. The relative abundance is on the y axis, while time is on the x axis.

Since, in the sequence, two lysine residues are present that could bind lactose, a mixture of the peptide with none, one or two lysine residues glycated was obtained (Figure 2).

In the figure above a peak corresponding to the di-lactosylated peptide is detected at 30.7 min, the mono-lactosylated peptide at 31.02 min and the unreacted peptide at 31.48 min. Peptide identification was obtained from the MS spectra. For the modified forms the identification was obtained considering an increase in peptide molecular weight of 324 for the mono-lactosylated form and 628 for the di-lactosylated form.

The mixture was then analysed with LTQ-Orbitrap high resolution mass spectrometry in order to verify which lysine residue was preferably involved in the glycation reaction.

**Figure 3:**
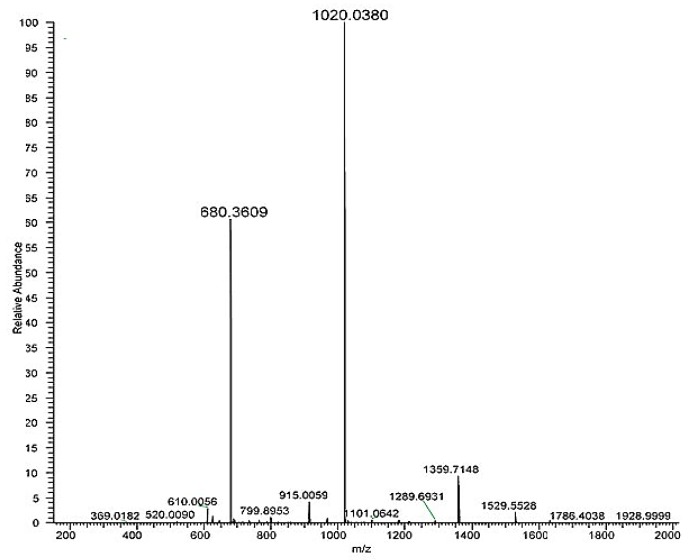
HRMS full scan spectra of the mono-lactosylated peptide. Relative abundance is on the y axis, mass-to-charge (*m*/*z*) ratio on the x axis.

**Figure A1:**
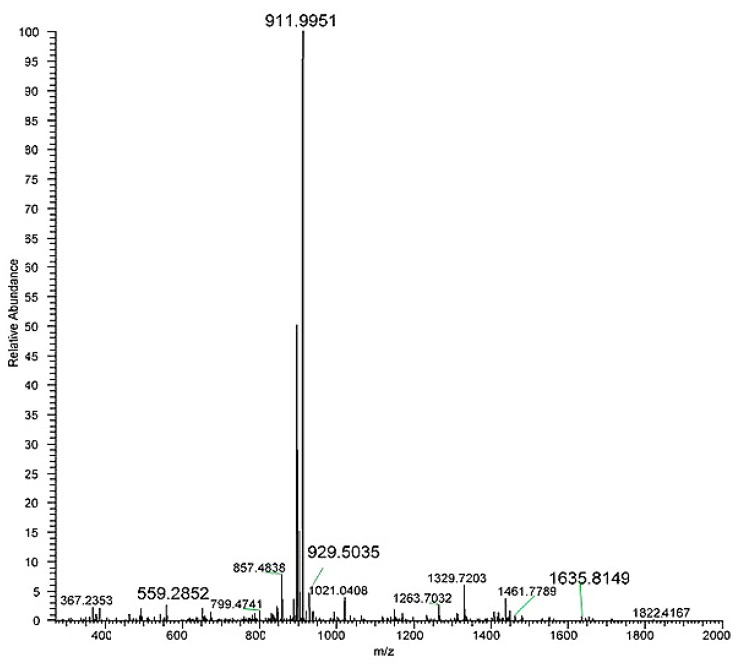
HRMS/MS spectrum obtained fragmenting the doubly charged ion of the mono-lactosylated peptide. Relative abundance is on the y axis, mass-to-charge (*m*/*z*) ratio on the x axis.

Analysing full scan spectrum (Figure 3) it was possible to identify MS ions corresponding to the mono-lactosylated peptide: 1020.0380 [M+2H]^2+^ and 680.3609 [M+3H]^3+^. The first listed MS ion was then fragmented (Figure A1 in the Appendix A).

**Figure 4:**
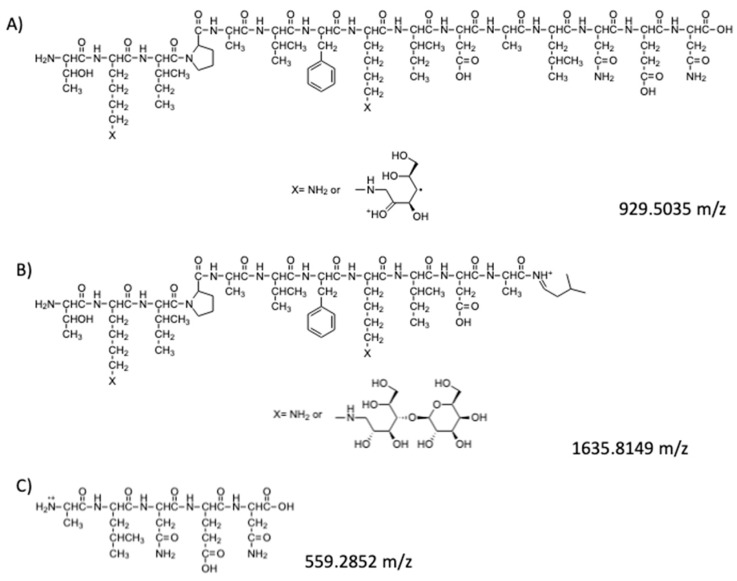
Peptides fragments identified in the HRMS/MS spectrum: (**A**) hypothesized structure for fragment at 929.5035 *m*/*z*; (**B**) hypothesized structure for fragment at 1635.8149 *m*/*z*; (**C**) hypothesized structure for fragment at 559.2852 *m*/*z*.

The hypothetical structures of fragments identified in the HRMS/MS spectrum and the corresponding MS ion are shown in Figure 4. The software Mass Frontier 5.1™ (Thermo Electron Xcalibur^®^) was used for the identification.

**Figure 5:**
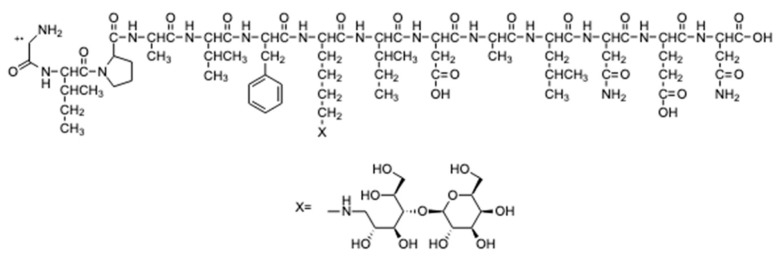
Peptide fragment identified in the HRMS/MS spectrum with only one lysine.

In addition to these fragments, the MS ion with higher intensity (911.9951 *m*/*z*) was identified in the HRMS/MS spectrum as the doubly charged ion of 1635.8149 [M+H]^+^ ion. The software indicates that this ion might correspond to a fragment of the mono-lactosylated peptide (Figure 5).

**Figure 6:**
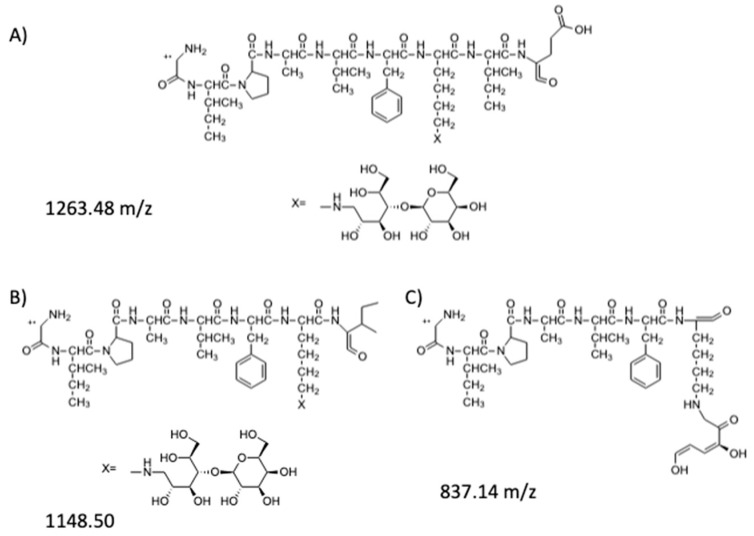
Fragments of the mono-lactosylated peptide identified in the MS^3^ spectrum: (**A**) hypothesized structure for fragment at 1263.48 *m*/*z*; (**B**) hypothesized structure for fragment at 1148.50 *m*/*z*; (**C**) hypothesized structure for fragment at 837.14 *m*/*z*.

**Figure A2:**
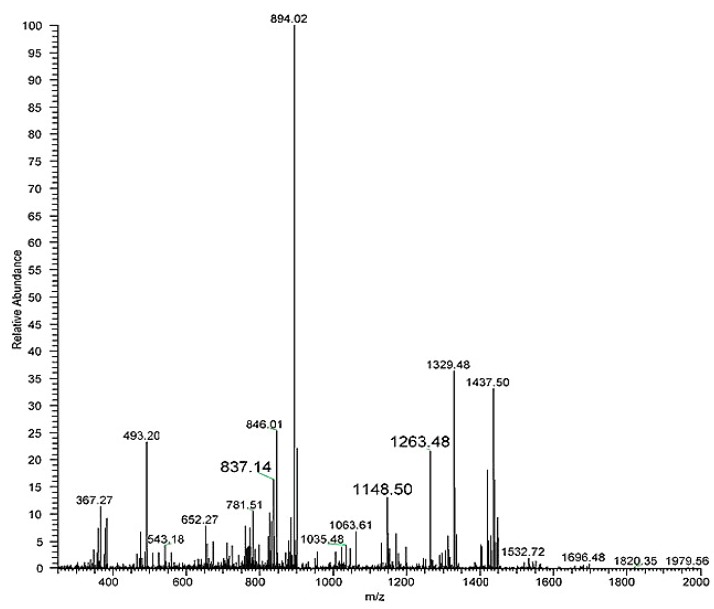
MS^3^ spectrum obtained from the fragmentation of the doubly charged ion identified in the HRMS/MS spectrum. Relative abundance is on the y axis, mass-to-charge (*m*/*z*) ratio on the x axis.

The sequence of this fragment presents only one lysine residue with a lactose bound to it. Thus, ion 911.9951 [M+2H]^2+^ was fragmented (the MS^3^ spectrum is reported in Figure A2 in the Appendix A) leading to some fragments (Figure 6) which confirm the hypothesised peptide structure shown in Figure 5.

The above data show that the lysine involved in the binding with lactose is the lysine in the middle of the sequence, between the residues of phenylalanine and isoleucine. Taking into consideration that the reaction was performed in solution with a large excess of lactose we can hypothesize that the above mentioned lysine residue should be more accessible for the lactosylation than the other. Thus, reaction conditions for the synthesis were modified to improve the yield of the mono-lactosylated peptide. Briefly, in the SPPS synthesis, the external lysine was protected with a Dde (*N*-[1-(4,4-dimethyl-2,6-dioxocyclohex-1-ylidene)ethyl]) group that remains bound to the residue also after cleavage. The reaction was performed in Dimethylformamide (DMF) [32]. Different reaction conditions were then used for optimizing yields (Table 8) which were determined by integration of the XICs peak areas before purification (MS ions are reported in Table 7) and dividing the area of the lactosylated peptide by the sum of the XIC areas of the modified and unmodified peptides.

Temperatures above 70 °C were found too high leading to the deprotection of the peptide. Molar ratio 1:25 peptide lactose in DMF was found too low and molar ratio higher than 1:50 did not induce better yields. Different reaction times were used (4 h, 24 h, 40 h, 48 h). After 48 h it was observed the presence of the deprotected peptide, thus the best reaction time was found 40 h. Hydrazine (2% in DMF) was used for the final deprotection of the Dde group. At this concentration lactose was partially removed, thus the final concentration was decreased to 1%. The deprotection was performed in a very short time in order to avoid the removal of lactose, and solvent was removed from the reaction mixture immediately after.

The mono-lactosylated peptide was finally obtained with the lactose bound on the desired residue at 90% yield. The developed protocol showed much higher yields as compared to analogous reactions reported in literature for the on-resin site-specific glycation of peptides with glucose in DMF (35% of yields) [30].

Such level of purity allows the use of peptide as probes for ELISA tests after a quick purification.

Future immunological studies will focus on the use of this peptide for studying the effect of lactosylation on IgE binding.

## 3. Materials and Methods

### 3.1. Reagents

Diethyl ether and Dimethylformamide (DMF) for peptide synthesis were purchased from Carlo Erba Reagents (Milan, Italy). Dichloromethane (DCM), N,N-diisopropylethylamine (DIPEA), Piperidine, Thioanisole, Triisopropylsilane (Tis), Dithiothreitol (DTT), Iodacetamide, Hydrochloric acid, β-lactoglobulin (98% of purity) and α-lactalbumin (92% of purity) standards, trypsin and α-chymotrypsin from bovine pancreas, Hydrazine monohydrate and the amino acid Fmoc-Lys(Dde)-OH were purchased by Sigma Aldrich (St. Luis, MO, USA). All the amino acids (Fmoc-Ala-OH, Fmoc-Asp(OtBu)-OH, Fmoc-Glu(OtBu)-OH, Fmoc-Ile-OH, Fmoc-Lys(Boc)-OH, Fmoc-Leu-OH, Fmoc-Asn(Trt)-OH, Fmoc-Val-OH, Fmo-Thr(tBu)-OH, Fmoc-Pro-OH, Fomc-Phe-OH, N,N,N’,N’-Tetramethyl-O-(1H-benzotriazol-1-yl)uranium hexafluorophosphate (HBTU) and the resin (Rink Amide MBHA resin 100–200 mesh) used for the synthesis were purchased from Novabiochem (Merk KGaA, Darmstadt, Germany). Trifluoroacetic acid (TFA) was purchased from Acros Organics (Thermo Fisher Scientific, Waltham, MA, USA). Formic acid was purchased from Fisher Scientific (Thermo Fisher Scientific, Waltham, MA, USA). The XT sample buffer, XT reducing agent 20x, Protein Standards, Criterion^TM^ XT 12% Bis-Tris precast gel, XT MES running buffer and Coomassie brilliant blue were purchased from BIO-RAD (Hercules, CA, USA). Quant-iT™ Protein Assay kit was purchased from Invitrogen (Thermo Fisher Scientific, Waltham, MA, USA). Doubly deionized water was obtained using a MilliQ system (Millipore, Bedford, MA, USA). HPLC grade Acetonitrile (ACN) was purchased from VWR International (Milan, Italy). Sep-Pak Plus C18 cartridges were purchased from Waters (Milford, MA, USA).

### 3.2. Whey Proteins Isolation

Different UHT (17) and pasteurized milk (7) samples were collected from the local retailers. A sample of raw milk was provided from a local farm. The extraction of the soluble whey proteins fraction was performed as follows: the fat part was removed centrifuging at 3220 g for 15 min at 4 °C and removing manually the fat layer from the upper side, the operation was repeated minimum three times. Caseins were then precipitated from the solution adjusting the pH to 4.6 with 0.5M HCl. After centrifugation at 3220 g for 15 min at 4 °C, supernatant whey was collected and filtered on 0.45 μm filters. Each sample was prepared in duplicate.

### 3.3. UPLC-MS Analysis

Whey protein samples were analysed with UPLC-MS analysis. The quantification of soluble whey proteins was performed by integration of the chromatographic XIC peaks areas and using two calibration curves, one for β-lactoglobulin and one for α-lactalbumin, prepared with standards of the two whey proteins at concentrations ranging from 0.0625 mM to 2 mM. All the samples were analysed in duplicate. 

An ACQUITY UPLC separation system with an Acquity UPLC^©^ Protein BEH C4 column (300 Å, 1.7 μm, 2.1 mm × 150 mm) was used to perform the UPLC-ESI-MS analysis. Eluent A was H_2_O + 0.1% HCOOH (eluent A) while eluent B CH_3_CN + 0.1% HCOOH (eluent B). The following steps were used for the gradient elution: isocratic 69% A for 7 min, from 69% A to 64.5% A by linear gradient in 13 min plus washing step at 100% B and reconditioning. Flow rate was set at 0.20 mL/min, sample temperature 18 °C, column temperature 35 °C and injection volume 4 μL. Waters SQ mass spectrometer was used for detection with the following conditions: ESI source in positive ionization mode, cone voltage 30V, capillary voltage 3.2 kV, desolvation temperature 300 °C, source temperature 150 °C, desolvation gas flow (N2): 650 L/h, cone gas flow (N2): 100 L/h. The software used for data processing was MassLynx^TM^ V4.0 (Waters Corporation, Milford MA, USA).

### 3.4. SDS-PAGE Analysis

For the SDS-PAGE analysis, the amount of sample needed was determined with the Quant-iT™ Protein Assay (Invitrogen, Thermo Scientific). Samples (nearly 40 μg of protein) were mixed with the XT sample buffer and the XT reducing agent. The marker was prepared mixing the protein standard, the XT sample buffer and the XT reducing agent. After 5 min at 95 °C and 5 min at −20°C, samples were loaded on a Criterion^TM^ XT Bis-Tris precast gel. Using an XT MES running buffer, gels were run for almost 60 min at constant voltage (150V). After the run, gels were stained with a Coomassie Blu solution (50% water MilliQ, 40% methanol, 10% Coomassie Brilliant Blue) for two hours in order to visualize protein bands. Gels were de-stained with a de-staining solution (50% MilliQ water, 40% Methanol, 10% Acetic Acid) for 20 min, repeating the operation for 3–4 times. Gels were then scanned using a GS-800 calibrated imaging densitometer (BIO-RAD).

### 3.5. In Solution Proteins’ Tryptic and Chymotryptic Digestion

Sample (5 mg) was dissolved in 200 μL of 50 mM NH_4_HCO_3_ and then 10 μL of DTT 200 mM (prepared in NH_4_HCO_3_ 100 mM) was added. After one hour at room temperature 8 μL of iodoacetamide 1M (prepared in NH_4_HCO_3_ 100 mM) were added and the solutions were stored at room temperature for one hour at the dark. Then 40 μL of DTT 200 mM was added. After one hour at room temperature chymotrypsin solution (2 mg of enzyme dissolved in 200 μL of NH_4_HCO_3_ 100 mM) is added at the ratio 1:50 enzyme:substrate. Samples were stored at 37 °C overnight and then purified through Sep-Pak C18 cartridges using eluent A (98% water, 2% acetonitrile and 0.1% formic acid) and eluent B (65% acetonitrile, 35% water and 0.1% formic acid).

LTQ-Orbitrap analysis was performed with a Jupiter^®^ 4 μm Proteo 90Å column (Phenomenex, 150 mm × 0.3 mm) and a μ-Precolumn™ Cartridge (Acclaim™ PepMap™ 100 C18, 5 μm, 100 Å, 300 μm × 5 mm). Eluent A was H_2_O + 0.2% HCOOH and eluent B was CH_3_CN + 0.2% HCOOH. For gradient elution it has proceeded as follows: isocratic 90% A for 4 min, from 90% A to 50% A by linear gradient in 56 min plus washing step at 95% B and reconditioning. Column temperature 35 °C. Samples were loaded using an enrichment cartridge with a loading flow of 30 μL/min (50% eluent A, 50% eluent B). The acquisition was performed in 5 steps: the first one was in full scan from 250 to 2000 *m*/*z* high resolution; from the second to the last step was in data-dependent scan. Fragmentation was performed in LTQ in CID mode and with collision energy 35. The software used was Xcalibur™ 2.0.7 (Thermo Fisher Scientific). For protein identification, the software used were Peaks^®^ (Bioinformatics solutions Inc, Waterloo ON, Canada) and Proteome Discoverer™ (Thermo Fisher Scientific, Waltham, MA, USA). Positive hits were arbitrary set for proteins identified with a score expressed as −10logP > 20 by the program. All the samples were prepared and analysed in duplicate.

Samples were also analysed with UPLC-MS analysis. It was performed with an ACQUITY UPLC^®^ separation system using an Acquity UPLC^©^ Protein BEH C18 (300Å, 1.7 μm, 2.1 mm × 150 mm) column with an ACQUITY UPLC^®^ Peptide CSH™ C18 VanGuard™ Pre-column (130Å, 1.7 μm, 2 mm × 5 mm). Eluent A was H_2_O + 0.1% HCOOH and eluent B was CH_3_CN + 0.1% HCOOH. Gradient elution was performed as follows: isocratic 100% A for 7 min, from 100% A to 53.5% A by linear gradient in 40 min, washing step at 100% B and reconditioning. Flow rate was set at 0.20 mL/min, sample temperature 18 °C, column temperature 35 °C and injection volume 4 μL. Detection was performed as described in Section 3.3. The MassLynx^TM^ V4.0 (Waters Corporation, Milford MA, USA) software was used for data processing. All samples were prepared and analysed in duplicate. For the identification of modified peptides in the MS spectra, the software Proteomics Toolkit (developed by the Institute for Systems Biology, Seattle, WA, USA) was also used.

### 3.6. Peptide Synthesis

Peptides synthesis was performed with the automated standard Fmoc protocol for the Solid Phase Peptide Synthesis (Fmoc-SPPS protocol). Peptides were synthesized using the Syro I automated synthesizer (Biotage). Peptides (TKIPAVFKIDALNEN and TK(Dde)IPAVFKIDALNEN) were obtained as amide at the C-terminus of the sequence loading manually a Rink Amide resin with Asparagine as first amino acid. The loading of the first amino acid was performed according to the following protocol. Swelling of the resin in DCM for 30 min. Fmoc deprotection with a 20% piperidine solution in DMF. Both these two steps were repeated twice, followed by DMF washes. The coupling reaction was performed suspending the resin in a DMF solution containing 5 equivalents of the amino acid, 4.8 equivalents of HBTU and 10 equivalents of DIPEA. It was left overnight under agitation then washed with DMF and DCM and dried under vacuum. Capping reaction was carried out using a solution of acetic anhydride 1:9 in DMF and, for 10 min under agitation. This step was repeated 2 times. Then resin was washed with 5% DIPEA in DMF (5 min under agitation) and 5% DIPEA in DCM (5 min under agitation), then resin was dried under vacuum.

Before the automated synthesis, the resin was swelled in DCM. The automated synthesis was planned according to the manufacturer standard protocol. Fmoc deprotection was performed using piperidine 40% in DMF. Amino acidic couplings were performed in DMF with 8 equivalents of DIPEA and 4 equivalents of amino acids and HBTU to the initial loading of the resin. Coupling takes 40 min. After each step of deprotection and coupling, several washes with DMF were introduced. At the end, the resin was washed with DCM and dried under vacuum. Before peptide cleavage from the resin, a final acetylation step was performed. Dry resin was then suspended in a mixture of TFA (95%), Tis (2.5%) and Thioanisole (2.5%) for the cleavage of the peptide from the resin. After 2 h the solution was recovered and the resin washed with TFA and ACN. The final solution was then dried under nitrogen flux. The dried peptide is stored overnight in diethyl ether at −20 °C. The peptide was then recovered by centrifugation, washed with diethyl ether and dried under vacuum. A small amount of peptide is dissolved in double deionized water with 0.1% of Formic Acid and analysed with UPLC-MS. The analytical column used was an Acquity BEH C18 (Waters, 300 A, 1.7 um, 2.1 mm × 150 mm). Eluent A was H_2_O+0.1% HCOOH and CH_3_CN + 0.1% HCOOH was eluent B. For gradient elution, the following steps were applied: isocratic 100% A for 7 min, from 100% A to 53.5% A by linear gradient in 40 min and 1 min at 53.5% A plus washing step 100% B and reconditioning. Flow rate was set at 0.20 mL/min, sample temperature 18 °C, column temperature 35 °C and injection volume 4 μL. Detection was performed as described in Section 3.5.

### 3.7. Peptide Purification and Quantification

Synthesized peptides were purified with Semi-preparative HPLC (Waters 1525 Binary HPLC Pump with a 998 detector, Waters). The column used was a Jupiter C18 column (250 mm × 10 mm, 300 Å, Phenomenex, Torrance, CA, USA). Eluent A was H_2_O + 0.1% of TFA, eluent B was CH_3_CN + 0.1% TFA. The UV absorption spectrum was set at 214 nm, flow rate at 4 mL/min. Eluents gradients were optimised for each peptide. Fractions, that were manually collected, were dried and analysed with UPLC-MS analysis (as described in Section 3.5) to verify peptides purity. The quantification was performed with HPLC-UV analysis (Waters Alliance 2695 separation module equipped with a dual λ absorbance detector 2487, Waters) using a Jupiter 5 μm C18 (250 × 2.0 mm, Phenomenex). Eluent A was water with 0.1% TFA, eluent B was acetonitrile with 0.1%. For gradient elution, the following steps were applied: isocratic 90% A for 5 min, from 90% A to 40% A by linear gradient in 50 min plus washing step at 100% B and reconditioning. Flow rate was set at 0.20 mL/min, injection volume 10 μL, column temperature 35 °C. Detection was performed at 214 nm. Quantification was obtained applying the Lambert-Beer equation and calculating the ε as reported in literature [33].

### 3.8. Conjugation of Peptide TKIPAVFKIDALNEN with Lactose

Dried peptide was homogenized with lactose (lysine:lactose ratio 1:16) in 10 mM buffer phosphate pH 8. The mixture was dried under nitrogen and heated at 70 °C for 48 h (Table 6, entry 4). Reaction mixture was then characterized with LTQ-Orbitrap high-resolution mass spectrometry as described in Section 3.5 and with UHPLC-MS/MS analysis as follow. An UHPLC system (Dionex Ultimate 3000, Thermo Scientific, Waltham, MA, USA) with a reverse phase column (Aeris Peptide 1.7 µm XB-C18, 150 × 2.10 mm, Phenomenex, Torrance, CA, USA) was used. Eluent A was H_2_O + 0.2% CH_3_CN + 0.1% HCOOH and eluent B was CH_3_CN + 0.2% H_2_O + 0.1% HCOOH. Flow was set at 0.2 mL/min and the gradient used was: 0–7 min, 100% A; 7–50 min, from 100% A to 50% A; 50–52.6 min, 50% A; 52.6–53 min, from 50% A to 0% A; 53–58.2 min, 0% A; 58.2–59 min, from 0% A to 100% A; 59–72 min, 100% A. Column temperature was 35 °C and sample temperature was 18 °C. Injection volume was 2 µL. A triple quadrupole TSQ Vantage (Thermo Scientific, Waltham, MA, USA) was used for detection applying the following parameters: acquisition time: 7–58.2 min, positive ion mode, acquisition range: 100–1500 *m*/*z*, micro scans: 1, Scan Time: 0.50, Q1 PW: 0.70, capillary temperature: 250 °C, spray voltage: 3200 V, sheath gas flow: 22 units, vaporizer temperature: 250 °C. Depending on the charge and mass of the ion to be fragmented, different collision energies (CE) were applied.

### 3.9. Conjugation of Peptide TK(Dde)IPAVFKIDALNEN with Lactose

Dried peptide was dissolved in DMF in a Pyrex glass tube. Lactose (1:50 molar ratio with the peptide) was dissolved in DMF and mixed with the peptide. The mixture was flushed under nitrogen and then heated at 70 °C for 48 h (Table 8, entry 11). After cooling down, it was added hydrazine to a final amount of 1% in DMF in the solution for the Dde deprotection. After 3 min under stirring, solvent was removed under vacuum. The reaction mixture was washed with doubly deionized water and purified with Sep-Pak C18 cartridges using eluent A (98% water, 2% acetonitrile and 0.1% formic acid) and eluent B (65% acetonitrile, 35% water and 0.1% formic acid). The obtained residue was then analysed with UPLC-MS analysis as described in Section 3.5.

## 4. Conclusions

The correlation between the harshness of the treatment with the degree of protein lactosylation was investigated on whey proteins, with a deep molecular characterization of the products. Comparing the data obtained after the UPLC-MS analysis in pasteurized and UHT treated samples, an increasing trend in proteins lactosylation due to the harshness of treatments was confirmed. After in solution tryptic and chymotryptic digestion, some lactosylation sites in both α-lactalbumin and β-lactoglobulin were identified. The comparison with known reported IgE-binding epitopes confirmed the presence of some modified lysine residues. This information is crucial in the study of the effects of this modification on protein allergenicity. Four β-lactoglobulin IgE-binding epitopes were synthesised with Fmoc Solid Phase Peptide Synthesis and a procedure for the in solution peptide lactosylation developed. The analysis of peptide TKIPAVFKIDALNEN lactosylation with LTQ-Orbitrap suggests a sort of preferential site in the sequence for the condensation of lactose with the lysine residue. Thus, a procedure for the selective lactosylation of a lysine residue was also developed. These epitopes synthesised in the unmodified and lactosylated forms will be used for further investigations, with ELISA tests, on the effects of lactose on the binding with human IgE.

## Figures and Tables

**Table 1:** Whey proteins quantification and percentages of lactosylated forms.

	α-lactalbumin	β-lactoglobulin
Milk sample	% of lactosylated forms	% of lactosylated forms
*Raw milk (n = 1)*	8	12
*Pasteurized milk (n = 7)*	6–7	15–16
*UHT milk (n = 17)*	12–36	26–52

**Table 2:** Glycation sites identified in α-lactalbumin after tryptic and chymotryptic digestion of whey samples from UHT milk.

Residue	Trypsin	Chimotrypsin
K5	-	Hexose
K13	-	Lactose/Hexose
K16	-	Hexose
K58	Lactose	Lactose/Hexose
K62	Lactose	Lactose
K79	Lactose/Hexose	-
K93	Hexose	-
K94	-	Lactose
K98	Lactose	-
K108	-	Hexose
K114	-	-
K122	Hexose	-

**Table 3:** Glycation sites identified in β-lactoglobulin after tryptic and chymotryptic digestion of whey samples from UHT samples.

Residue	Trypsin	Chimotrypsin
K8	-	-
K14	Lactose	Lactose
K47	Lactose/Hexose	Lactose
K60	-	-
K69	Lactose	-
K70	Hexose	Lactose
K75	Hexose	-
K77	Hexose	-
K83	Lactose	Lactose
K91	Lactose	-
K100	Hexose	-
K101	Hexose	Lactose/Hexose
K135	Lactose	Lactose/Hexose
K138	-	Lactose/Hexose
K141	Hexose	Lactose

**Table 4:** Identified modified peptides deriving from β-lactoglobulin in the chymotryptic and tryptic digestions (in bold is highlighted the modified residue).

Peptide	Modification	MS Ions
**K**IDALNENKVL	Lactosylation	1581 [M+H]^+^ 791 [M+2H]^2+^ 473.7 (Y4)^+^ 132.1 (Y1)^+^
TPEVDDEALE**K**FDK	Lactosylation	980.6 [M+2H]^2+^ 654.1 [M+3H]^3+^
WENGECAQ**K**	Carboxymethyl lysine	1179.5 [M+H]^+^ 590.4 [M+2H]^2+^
IDALNEN**K**VLVLDTDYK	Lactosylation	1145.0 [M+2H]^2+^ 763.1 [M+3H]^3+^
VLVLDTDY**K**K	Lactosylation	1518.5 [M+H]^+^ 759.6 [M+2H]^2+^
VRTPEVDDEALE**K**F	Lactosylation	986.8 [M+2H]^2+^ 658.2 [M+3H]^3+^ 166.0 (Y1)^+^

**Table 5:** β-lactoglobulin IgE-binding epitopes reported in literature [26] that have lactosylation sites (highlighted in bold).

IgE-binding Epitope
TKIPAVF**K**IDALNEN
TKIPAVF**K**IDALNEN**K**VLVL
CLVRTPEVDDEALE**K**FD**K**AL
LE**K**FD**K**ALKALPMHIRLSFN

**Table 6:** Reaction conditions tested for peptide lactosylation.

	Peptide:Lactose Molar Ratio	Temperature (°C)	Reaction Time (hours)	Reaction Yields *
1	1:4	60	20	27%
2	1:8	60	20	29%
3	1:12	70	48	25%
4	1:16	70	48	39%

* yields are expressed as percentages of mono lactosylated peptide on the total amount of peptide (underivatized and lactosylated).

**Table 7:** MS ion of the synthesised peptide identified from the UPLC-MS spectra.

	MS Calculated Ions	MS Identified Ions
Unreacted Dde protected peptide *(retention time 38.9 min)*	1879.15 [M+H]^+^; 940.07 [M+2H]^2+^; 627.05 [M+3H]^3+^; 470.54 [M+4H]^4+^	1878.7 [M+H]^+^; 940.1 [M+2H]^2+^; 627.2 [M+3H]^3+^
Lactosylated Dde protected peptide *(retention time 38.07 min)*	2203.45 [M+H]^+^; 1102.23 [M+2H]^2+^; 735.16 [M+3H]^3+^; 551.62 [M+4H]^4+^	1102.2 [M+2H]^2+^; 735.2 [M+3H]^3+^; 550.6 [M+4H]^4+^

**Table 8:** Reaction conditions tested in the optimization of the site-specific peptide lactosylation.

	Peptide/Lactose Molar Ratio	Solvent	Temperature (°C)	Reaction Time (hours)	Reaction Yields
1	1:12	Dry	70	48	22%
2	1:24	Dry	70	48	19%
3	1:24	Dry	80	48	23%
4	1:50	Dry	70	48	21%
5	1:100	Dry	70	48	42%
6	1:70	Dry	70	48	46%
7	1:25	DMF	70	24	74%
8	1:25	DMF	70	48	61%
9	1:50	DMF	70	4	63%
10	1:50	DMF	70	24	80%
11	1:50	DMF	70	48	90%

For the identification of the lactosylation sites the analysed modifications were lactosylation, glycation with a hexose and carboxymethylation (marker of the advanced Maillard reaction).

## Supplementary Materials

The following are available online at https://www.mdpi.com/1420-3049/25/6/1294/s1, Table S1: List of the identified α-lactalbumin peptides with glycated lysine residues after tryptic and chymotryptic in solution digestion, Table S2: List of the identified β-lactoglobulin peptides with glycated lysines after tryptic and chymotryptic in solution digestion.
