# Supplementary material for: Thermally-Induced Lactosylation of Whey Proteins: Identification and Synthesis of Lactosylated β-lactoglobulin Epitope
Source: Molecules. 2020 Mar 12;25(6):1294. doi: 10.3390/molecules25061294 (PMC7143954; doi:10.3390/molecules25061294)

Supplementary material

Table S1. List of the identified α-lactalbumin peptides with glycated lysine residues after tryptic and chymotryptic *in solution* digestion.

Trypsin	Chymotrypsin
QINNKIW	TKCEVFRELKDL
IWCKDDHNPHSSNICNISCDK	TKCEVFREL
DDHNPHSSNICNISCDK	RELKDL
FLDDDLTDDIMCVKK	KDLKGYGGVSL
DKFLDDDLTDDIM	QINNKIW
ILDKVGINY	IWCKDDHNPHSSNICNISCDK
LDQWLCEK	CKDDHNPHSSNICNISCDKFL
	KILDKVGINY
	LAHKALCSEKLDQW
	LAHKAL

For the identification of the glycation sites the analysed modifications were: lactosylation, glycation with a hexose and carboxymethylation (marker of the advanced Maillard reaction).

Table S2. List of the identified β-lactoglobulin peptides with glycated lysines after tryptic and chymotryptic *in solution* digestion.

Trypsin	Chymotrypsin
LIVTQTMKGLDIQK	DIQKVAGTW
GLDIQKVAGTWY	VEELKPTPEGDLEIL
VYVEELKPTPEGDLEILLQK	VEELKPTPEGDLEILLQK
VYVEELKPTPEGDLEILLQKWENGECAQKKIIAEK	VEELKPTPEGDLEILLQ
VYVEELKPTPEGDLEIL	VEELKPTPEGDLEILL
WENGECAQK	WENGECAQKK
WENGECAQKK	ENGECAQKKIIAEKTKIPAVF
IIAEKTK	IIAEKTK
IIAEKTKIPAVFK	KIDALNENKVL
IDALNENKVLVLDTDYK	KIDALNENKVLVL
IDALNENKVL	VLVLDTDYKKY
KIDALNENKVL	DTDYKKYLLF
VLVLDTDYKK	VRTPEVDDEALEK
TPEVDDEALEKFDK	VRTPEVDDEALEKF
TPEVDDEALEKF	VRTPEVDDEALEKFDKAL
FDKALK	TPEVDDEALEKF
ALKALPMHIR	EKFDKALKAL

For the identification of the glycation sites the analysed modifications were: lactosylation, glycation with a hexose and carboxymethylation (marker of the advanced Maillard reaction).

Figure S1. SDS-PAGE gel image (1,2 = pasteurized milk; 3,4 = UHT milk; p = precipitated caseins; s = extracted whey). Marker values are expressed as kDa.

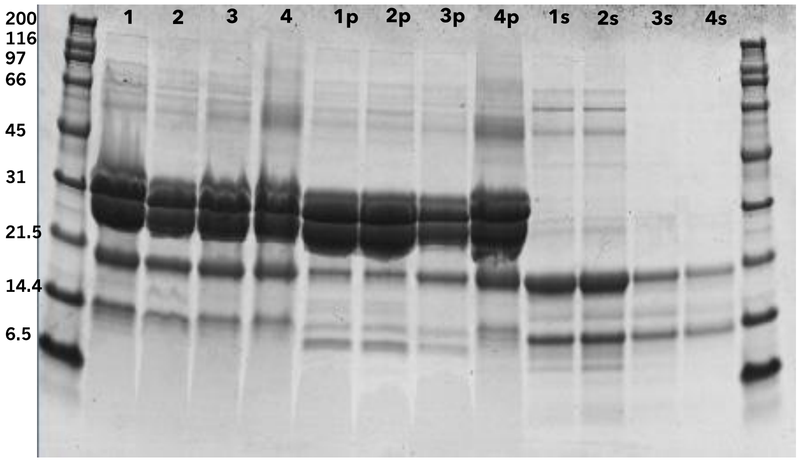

Supplement: Supplementary file 1 [file molecules-25-01294-s001.pdf]
